# Supplementary material for: Characterization of Transcriptome Dynamics during Early Fruit Development in Olive (Olea europaea L.)
Source: Int J Mol Sci. 2023 Jan 4;24(2):961. doi: 10.3390/ijms24020961 (PMC9864153; doi:10.3390/ijms24020961)
Supplement: Supplementary file 1 [file ijms-24-00961-s001.zip › ijms-2085432-supplementary.pdf]

Characterization of transcriptome dynamics during early fruit development in olive (*Olea europaea* L.)

Maria C. Camarero, Beatriz Briegas, Jorge Corbacho, Juana Labrador, Mercedes Gallardo, Maria C. Gomez-Jimenez*

***Correspondence:**
Maria C. Gomez-Jimenez
mcgomez@unex.es

SUPPLEMENTARY MATERIAL

Table S1. Summary of the mapping quality parameters.

Sample	Mapped reads	Properly Pair	%Properly Pair	HQ Reads	%HQ Reads
**P0_1**	37017193	18587998	50.21	27034825	73.03
**P14_1**	42736280	23493118	54.97	30931853	72.38
**P28_1**	41715362	24299272	58.25	33419141	80.11
**P0_2**	37520924	24140268	64.34	30815628	82.13
**P14_2**	40164262	24780862	61.70	32549821	81.04
**P28_2**	42297550	25035646	59.19	34052422	80.51
**P0_3**	39025290	22490878	57.63	31518579	80.76
**P14_3**	41435922	24547362	59.24	30692496	74.07
**P28_3**	42184150	23795280	56.40	34843085	82.59

Table S2. Genes up-regulated during early olive fruit development in the first comparison (P14 versus P0). (XLS)

Table S3. Genes down-regulated during early olive fruit development in the first comparison (P14 versus P0). (XLS)

Table S4. Genes up-regulated during early olive fruit development in the second comparison (P28 versus P14). (XLS)

Table S5. Genes down-regulated during early olive fruit development in the second comparison (P28 versus P14). (XLS)

Table S6. Genes up-regulated during early olive fruit development in both the first (P14 versus P0) and second (P28 versus P14) comparisons. (XLS)

Table S7. Genes down-regulated during early olive fruit development in both the first (P14 versus P0) and second (P28 versus P14) comparisons. (XLS)

Table S8. Specific or exclusively expressed genes in olive fruit at selected stage during early fruit development. (XLS)

Table S9. The enrichment analysis of GO terms based on up-regulated DEGs in the dividing fruits at 14 (P14) versus the flowers at anthesis stage (P0) during early olive fruit development. (XLS)

Table S10. The enrichment analysis of GO terms based on down-regulated DEGs in the dividing fruits at 14 (P14) versus the flowers at anthesis stage (P0) during early olive fruit development**.** (XLS)

Table S11. The enrichment analysis of GO terms based on up-regulated DEGs in the expanding fruits at 28 (P28) DPA versus the dividing fruits at 14 (P14) during early olive fruit development. (XLS)

Table S12. The enrichment analysis of GO terms based on down-regulated DEGs in the expanding fruits at 28 (P28) DPA versus the dividing fruits at 14 (P14) during early olive fruit development. (XLS)

Table S13. Cell-cycle-related genes induced or repressed in the dividing fruits at 14 (P14) versus the flowers at anthesis stage (P0), and the expanding fruits at 28 (P28) DPA versus the dividing fruits at 14 (P14) during early olive fruit development. (XLS)

Table S14. Hormone-related genes induced or repressed in the dividing fruits at 14 (P14) versus the flowers at anthesis stage (P0), and the expanding fruits at 28 (P28) DPA versus the dividing fruits at 14 (P14) during early olive fruit development. (XLS)

Table S15. Peptide-signaling-related and subtilisin-like proteases genes induced or repressed in the dividing fruits at 14 (P14) versus the flowers at anthesis stage (P0), and the expanding fruits at 28 (P28) DPA versus the dividing fruits at 14 (P14) during early olive fruit development. (XLS)

Table S16. Proteases genes induced or repressed in the dividing fruits at 14 (P14) versus the flowers at anthesis stage (P0), and the expanding fruits at 28 (P28) DPA versus the dividing fruits at 14 (P14) during early olive fruit development. (XLS)

Table S17. Cell-wall-related genes induced or repressed in the dividing fruits at 14 (P14) versus the flowers at anthesis stage (P0), and the expanding fruits at 28 (P28) DPA versus the dividing fruits at 14 (P14) during early olive fruit development. (XLS)

Table S18. Transport-related and vesicle-trafficking-related genes repressed or induced in the dividing fruits at 14 (P14) versus the flowers at anthesis stage (P0), and the expanding fruits at 28 (P28) DPA versus the dividing fruits at 14 (P14) during early olive fruit development. (XLS)

Table S19. Transcription factors (TF) genes repressed or induced in the dividing fruits at 14 (P14) versus the flowers at anthesis stage (P0), and the expanding fruits at 28 (P28) DPA versus the dividing fruits at 14 (P14) during early olive fruit development. (XLS)

Table S20. PCR-primers used in this study.

**Primer**	**Sequence**	**Gene_ID**
CS2-F	5’-ATGACTGGTTTTCAGGACTAC-3’	*OeCS2* (XP_022870513.1)
CS2-R	5’-AAATTCAACTGTAGAGGACGT-3’	
DIR15-F	5’-AAACCGAGCGAACCGACCCCT-3’	*OeDIR15* (XP_022897725.1)
DIR15-R	5’-AACCACCGCCATTTCTCTACC-3’	
EXPB2-F	5’-GGTTCCAGTGACTACTATTT-3’	*OeEXPB2* (XP_022856136.1)
EXPB2-R	5’-CAATGGATTTTCTGAATTGAG-3’	
COX-6A-F	5’- GGCGGCGCATCTACTCCACGCTCC-3’	*OeCox-6A* (XP_027178774.1)
COX-6A-R	5’- ACCCTTGGAAAGGTTAATGACTGC -3’	
CYCA2;1-F	5’-CCATCATCCTACGATCCATGC-3’	*OeCYCA2;1* (XP_022881536.1)

CYCA2;1-R	5’-TGCAGAGGAACTTGCACCAGG-3’	
CYCA3;1-F	5’-AGCTATGCGT GAGATTCTGGT-3’	*OeCYCA3;1* (XP_022860599.1)
CYCA3;1-R	5’-TCCATGTGTATAACGTCTTCT-3’	
CYCC1;1-F	5’-TGCTGCCACATCCATTTGCC-3’	*OeCYCC1;1* (XP_022874690.1)
CYCC1;1-R	5’- ACATTTTGAGCCAATCTTGC-3’	
CYCB2;4-F	5’-TTGGTAGACTGCCTCATTGT-3’	*OeCYCB2;4* (XP_022881380.1)
CYCB2;4-R	5’-GTCGAACTGCTGTTCCATAT-3’	
CYCB3;1-F	5’-GGAAAACGCTACTCTCTCAAG-3’	*OeCYCB3;1* (XP_022877828.1)
CYCB3;1-R	5’-TTGAGCTGATAACTTCCTCG-3’	
CYCB2;3-F	5’-AAATCGGACATATCCCCTGT-3’	*OeCYCB2;3* (XP_022895161.1)
CYCB2;3-R	5’-TTCTCATCTGATCTACGCAT-3’	
CYCU1;1-F	5’-AACAACACCTAGAGTTCTTACC-3’	*OeCYCU1;1* (XP_022845538.1)
CYCU1;1-R	5’-GATACTTATGCACCAACCTGT-3’	
CYCA1;4-F	5’-TACATAGATAATAATGAAAT-3’	*OeCYCA1;4* (XP_022875899.1)
CYCA1;4-R	5’-TCCATGAAGTCAGTAGTAGG-3’	
CDKC1-F	5’-CATGATTTGACTGGCCTTGCT-3’	*OeCDKC1* (XP_022880663.1)
CDKC1-R	5’-GATTAGTAAGATTAGCATTGTG-3’	
CDKB1-F	5’-CCGTTGCAGGAGGAAGAGAA-3’	*OeCDKB1* (XP_022860837.1)
CDKB1-R	5’-TCCATTATTTGATGTGGAT-3’	
CDKF1-F	5’-CAATCTCATATAAAAGTTGC-3’	*OeCDKF1* (XP_022893380.1)
CDKF1-R	5’-CGCTCGTGGA2TTTCGTACTT-3’	
CKI7-F	5’-TAGAACATCAGCTGCTTTCGA-3’	*OeCKI7* (XP_022844706.1)
CKI7-R	5’-TTTAGCTGAACTGTGGACCT-3’	
SMR6-F	5’-GGTACCCTTCTGTTTAAAAG-3’	*OeSMR6* (XP_022865500.1)
SMR6-R	5’-CGAACGGACGTCTTCATCTT-3’	
SMR9-F	5’-CCTGTAGAAGAAGAACCAGA-3’	*OeSMR9* (XP_022899245.1)
SMR9-R	5’-AATACCGTTGGTGTTGGGGT-3’	

ERFA-F	5'-AATACCGTTGGTGTTGGGGT-3'	*OeERFA* (XP_022841835.1)
ERFA-R	5'-TTTGTGACCTTTGACCTGCT-3'	
MYB3R1-F	5'-TACAGTCCACTTGGCATTCGC-3'	*OeMYB3R1* (XP_022853377.1)
MYB3R1-R	5'-ATGATGACAGCATAAGTACT-3'	
RBR3-F	5'-GCAGTGGGTGAATTATGGTT-3'	*OeRBR3* (XP_022889026.1)
RBR3-R	5'-GATTCTATATTCTACTACTC-3'	
SCL28-F	5'-GAGAAGTGAATCTCATAGTA-3'	*OeSCL28* (XP_022846070.1)
SCL28-R	5'-ATGATCATCGGATTCCTTAA-3'	
SBT1.5-F	5'-TCCTTCAAATTAAATCTCATC-3'	*OeSBT1.5* (XP_022854658.1)
SBT1.5-R	5'-ATTAGAGCCCGAATCAGACTC-3'	
SBT3.5-F	5'-CTCTCTTCAGTCCTGGGAAGT-3'	*OeSBT3.5* (XP_022844531.1)
SBT3.5-R	5'-CGATGAATTGAATGGTTCTCC-3'	
SBT3.6-R	5'-ATTCTTGGAGGCAAAAAGACC-3'	*OeSBT3.6* (XP_022853451.1)
SBT3.6-R	5'-AGCACCATATTGACCTCTCAA-3'	
SBT1.8-F	5'-ATGGAGTCGAGTTCGGCTATT-3'	*OeSBT1.8* (XP_022845741.1)
SBT1.8-R	5'-GAAACCATGGTACGCTGTGTC-3'	
SBT1.1-F	5'-GCAGAAAGAGAAACATATGTG-3'	*OeSBT1.1* (XP_022859421.1)
SBT1.1-R	5'AGCATTCCACAGGCCACTACC-3'	

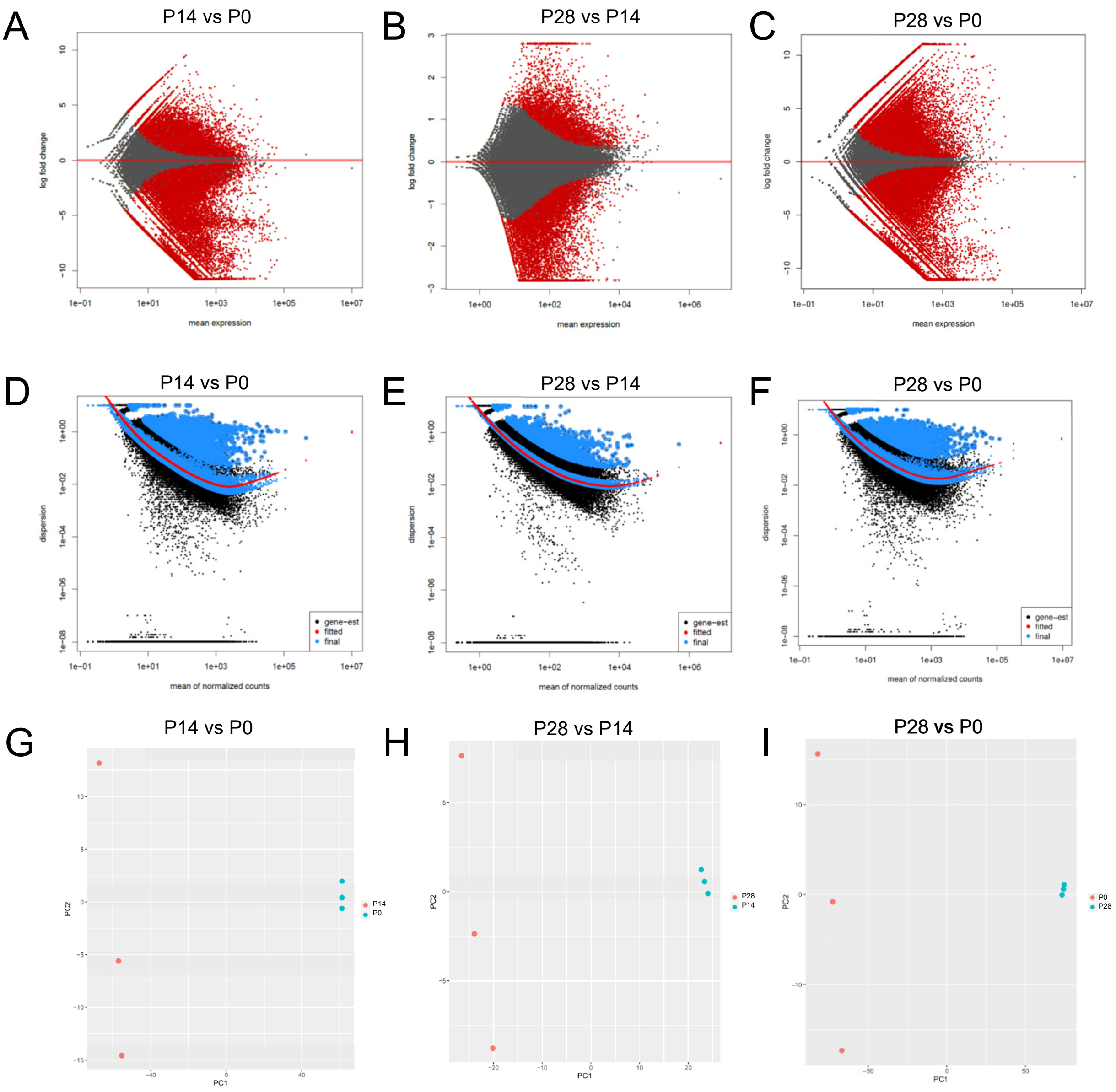

Figure S1. Whole transcripts expression during early development in 'Picual' olive. **(A)** MA plot for differential expression analysis for each gene, the log_2 (fold change) (log_2(P14/P0) between fruit at 14 DPA and at 0 DPA samples is plotted (A, y axis) against the gene's log_2 (average expression) (M, x axis). **(B)** MA plot for differential expression analysis for each gene, the log_2 (fold change) (log_2(P28/P14) between fruit at 28 DPA and at 14 DPA samples is plotted (A, y axis) against the gene's log_2(average expression) (M, x axis). **(C)** MA plot for differential expression analysis for each gene, the log_2 (fold change) (log_2(P28/P0) between fruit at 28 DPA and at 0 DPA samples is plotted (A, y axis) against the gene's log_2(average expression) (M, x axis). Transcripts that are identified as significantly differentially expressed are coloured in red **(D)**Dispersion plot for differential expression analysis between P14 and P0 samples (P14 versus P0). **(E)** Dispersion plot for differential expression analysis between P28 and P14 samples (P28 versus P14). **(F)** Dispersion plot for differential expression analysis between P14 and P0 samples (P28 versus P0) **(G)** Principal component analysis of expression data between P14 and P0 samples (P14 versus P0). **(H)** Principal component analysis of expression data between P28 and P14 samples (P28 versus P14). **(I)** Principal component analysis of expression data between P28 and P0 samples (P28 versus P0).

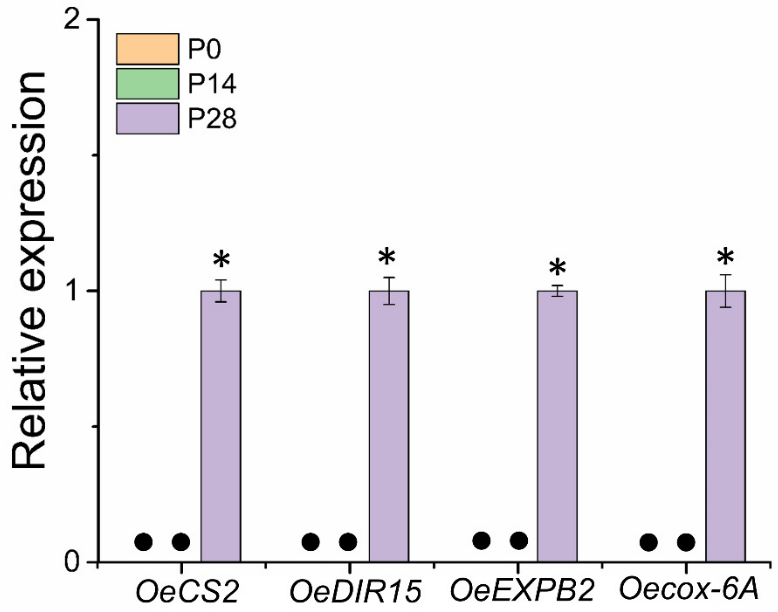

Figure S2. Expression of *OeCS2, OeDIR15, OeEXPB2* and *Oecox-6A* during early fruit development in olive. Data are the means ± SD of three biological replicates with three technical repeats each and were determined by qRT-PCR normalized against *Olea europaea* ubiquitine (Gomez-Jimenez et al., 2010). Statistically significant differences based on unpaired Student's *t*-test at $p < 0.05$ are denoted by an asterisk. Gene expressions not detected are denoted by a circle.

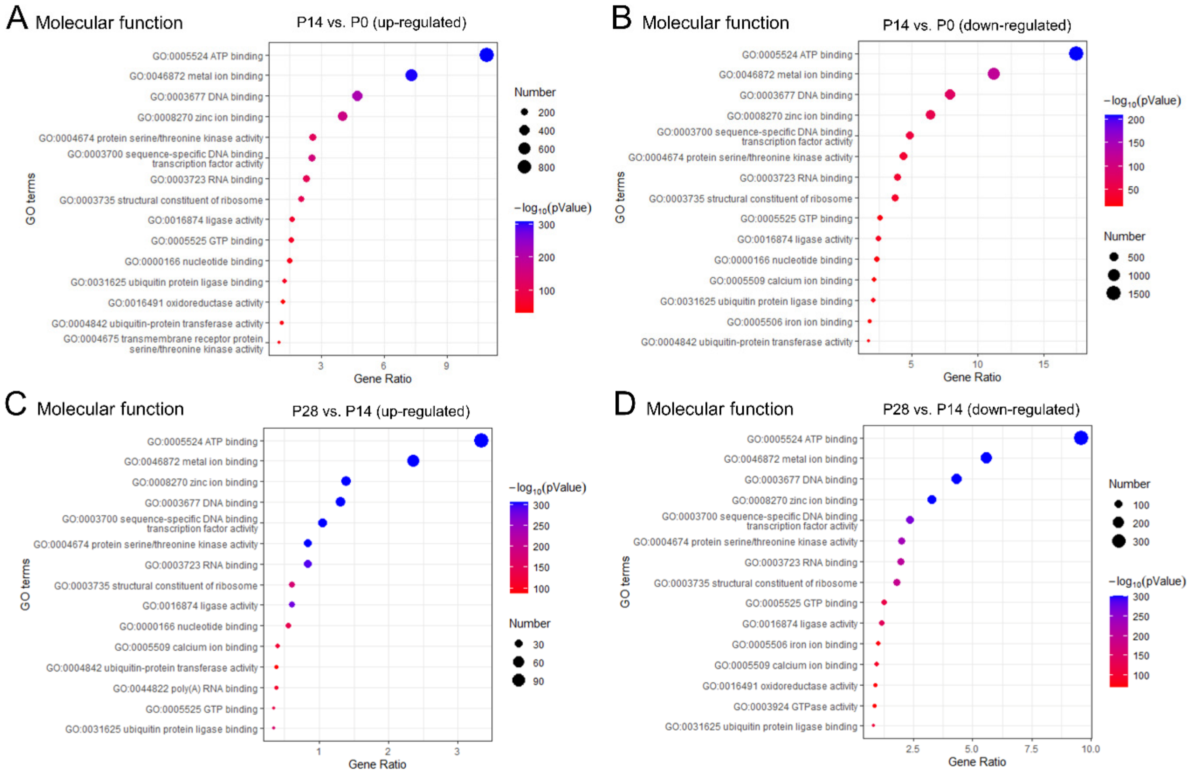

Figure S3. Functional analysis of DEGs during early fruit development in olive. The Top 15 enrichment analysis of GO 'molecular function' terms based on DEGs in olive flowers at anthesis stage (P0), and the developing fruits at 14 (P14) and 28 (P28) DPA. (Top 15). **(A)** Bubble Plot of GO 'molecular function' terms in the GO annotations of the genes of the 8040 transcripts with increased transcript accumulation, **(B)** and of the genes of the 8903 transcripts with decreased transcript accumulation in the P14 versus. P0 comparison; and **(C)** Bubble Plot of GO 'molecular function' terms in the GO annotations of the genes of the 3443 transcripts with increased transcript accumulation, and **(D)** of 3782 transcripts with decreased transcript accumulation in the P28 versus. P14 comparison. The Y-axis and X-axis denote GO name and gene ratio, respectively. The color of each bubble represents -$\log_{10}$ (p value), and each bubble's size of represents the count of DEGs. Additional information is presented in Tables S9-S12.

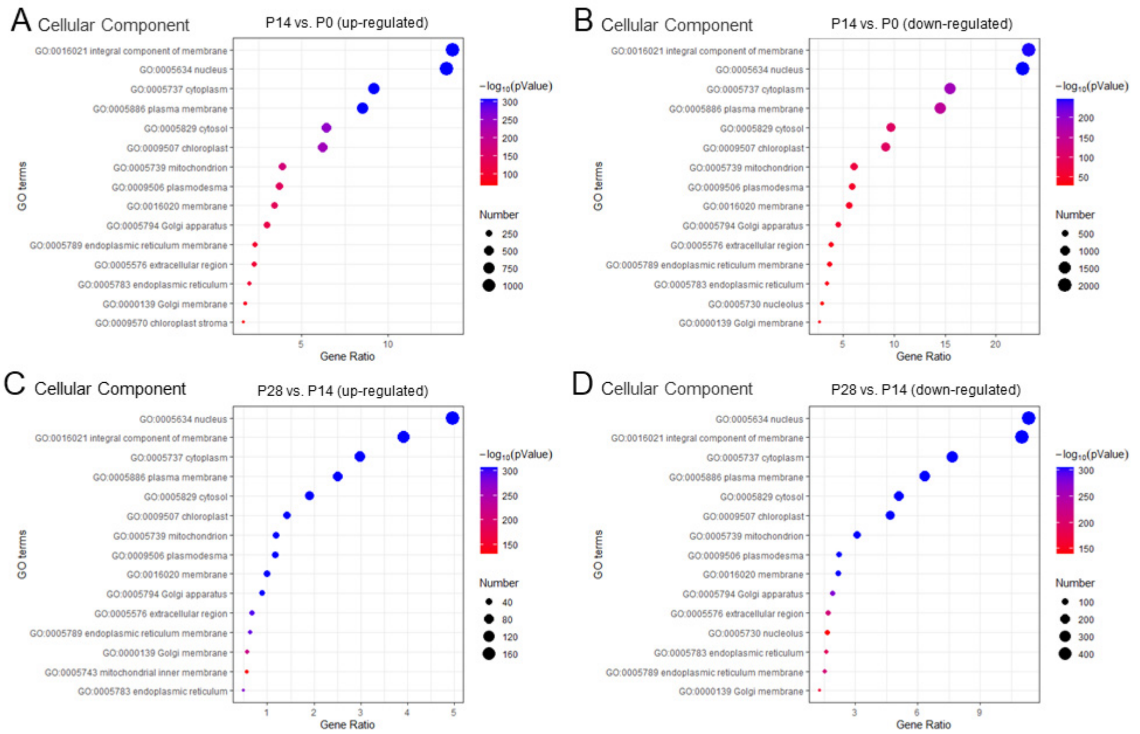

Figure S4. Functional analysis of DEGs during early fruit development in olive. The Top 15 enrichment analysis of GO 'cellular component' terms based on DEGs in olive flowers at anthesis stage (P0), and the developing fruits at 14 (P14) and 28 (P28) DPA. (Top 15). **(A)** Bubble Plot of GO 'cellular component' terms in the GO annotations of the genes of the 8040 transcripts with increased transcript accumulation, **(B)** and of the genes of the 8903 transcripts with decreased transcript accumulation in the P14 versus. P0 comparison; and **(C)** Bubble Plot of GO 'cellular component' terms in the GO annotations of the genes of the 3443 transcripts with increased transcript accumulation, and **(D)** of 3782 transcripts with decreased transcript accumulation in the P28 versus. P14 comparison. The Y-axis and X-axis denote GO name and gene ratio, respectively. The color of each bubble represents -$\log_{10}$ (p value), and each bubble's size of represents the count of DEGs. Additional information is presented in Tables S9-S12.

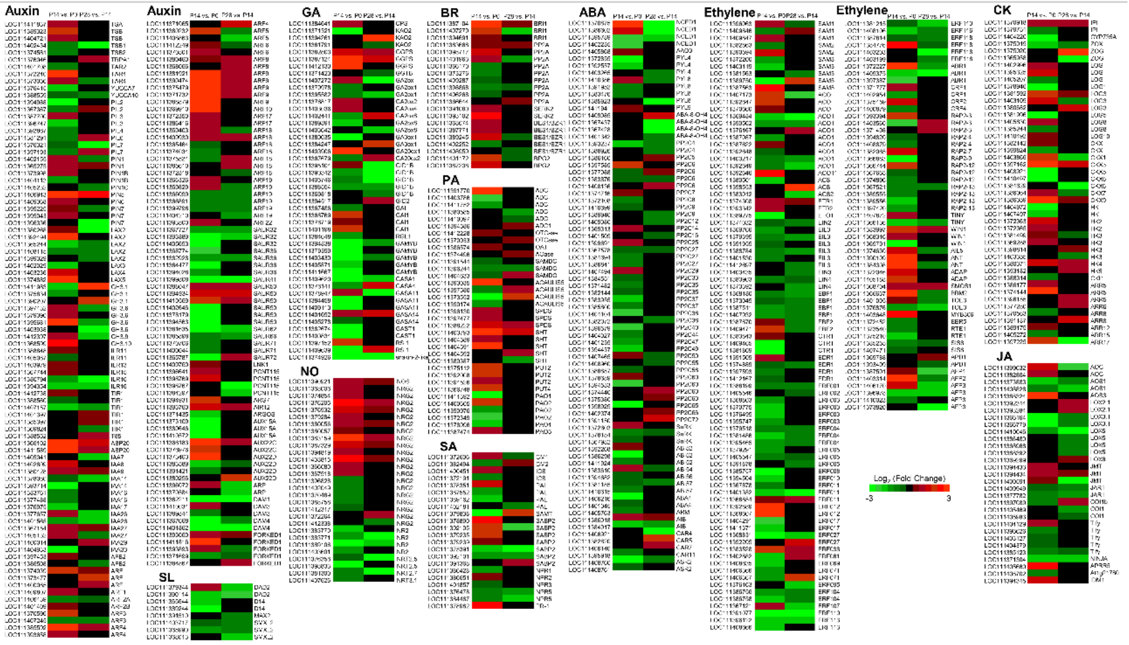

Figure S5. Differential gene expression of hormone-related genes during early olive fruit development. Expression values are represented in a heatmap as Log_2 Fold Change in both the P14 versus P0 (P14 vs. P0), and the P28 versus P14 (P28 vs. P14) comparisons, and the colour key is indicated at the bottom. Additional information on the hormone-related genes is presented in Table S14.
